# An efficient technique for Bayesian modeling of family data using the BUGS software

**DOI:** 10.3389/fgene.2014.00390

**Published:** 2014-11-18

**Authors:** Harold T. Bae, Thomas T. Perls, Paola Sebastiani

**Affiliations:** ^1^School of Biological and Population Health Sciences, College of Public Health and Human Sciences, Oregon State UniversityCorvallis, OR, USA; ^2^New England Centenarian Study, Department of Medicine, Boston University School of MedicineBoston, MA, USA; ^3^Department of Biostatistics, Boston University School of Public HealthBoston, MA, USA

**Keywords:** BUGS, parameterization, family-based study, covariance matrix, linear mixed models

## Abstract

Linear mixed models have become a popular tool to analyze continuous data from family-based designs by using random effects that model the correlation of subjects from the same family. However, mixed models for family data are challenging to implement with the BUGS (Bayesian inference Using Gibbs Sampling) software because of the high-dimensional covariance matrix of the random effects. This paper describes an efficient parameterization that utilizes the singular value decomposition of the covariance matrix of random effects, includes the BUGS code for such implementation, and extends the parameterization to generalized linear mixed models. The implementation is evaluated using simulated data and an example from a large family-based study is presented with a comparison to other existing methods.

## 1. Introduction

Many observational studies are designed using some form of clustered sampling that introduces correlation between observations within the same cluster. A popular study design that produces correlated observations is the family-based study (Ott et al., 2011), in which families (or clusters) may be selected because family members are enriched of some particular trait of interest. In this design, multiple relatives within the same family are enrolled in the study, and subjects from the same family cannot be assumed independent because they share some genetic background and may have more similar phenotypes than members from different families. In this context, standard statistical methods that assume *independent and identically distributed observations* are not appropriate because ignoring the correlation between observations may impact the false positive rates of statistical methods (Cannon et al., 2001).

When the trait of interest is continuous, a linear mixed-effects model can be used to account for the family structure by using random effects with a variance-covariance matrix that describes the within and between family covariances. However, the implementation of such models in the BUGS software (Lunn et al., 2000) becomes challenging due to the high dimensionality of the random effects vector, which is as large as the sample size in the study. The fact that the high-dimensional covariance matrix can only be updated as a composite whole in BUGS (Burton et al., 1999) increases the computational burden of the Markov Chain Monte Carlo (MCMC) estimation and often results in a failure to compile the model. To tackle this implementation issue, Waldmann (2009) have proposed an approach based on a decomposition of the multivariate normal distribution of the random effects into univariate normal distributions using conditional distributions (Hallander et al., 2010). Our experience with this approach is that it fails to produce accurate results with large multigenerational families.

This paper describes an alternative parameterization that uses the singular value decomposition of the large covariance matrix of the random effects to fit a mixed model with independent random effects and avoids the use of large variance matrices. This approach is not novel and, for example, it was used in factored spectrally transformed linear mixed models (FaST-LMM) (Lippert et al., 2011) for fast computations with family data. The novelty of our contribution is to use this approach for an efficient implementation in the BUGS software and to extend it to generalized linear models. The BUGS code is also provided and a specific example as well as results from simulation studies are presented with some discussion.

## 2. Proposed parameterization

**Figure 1:**
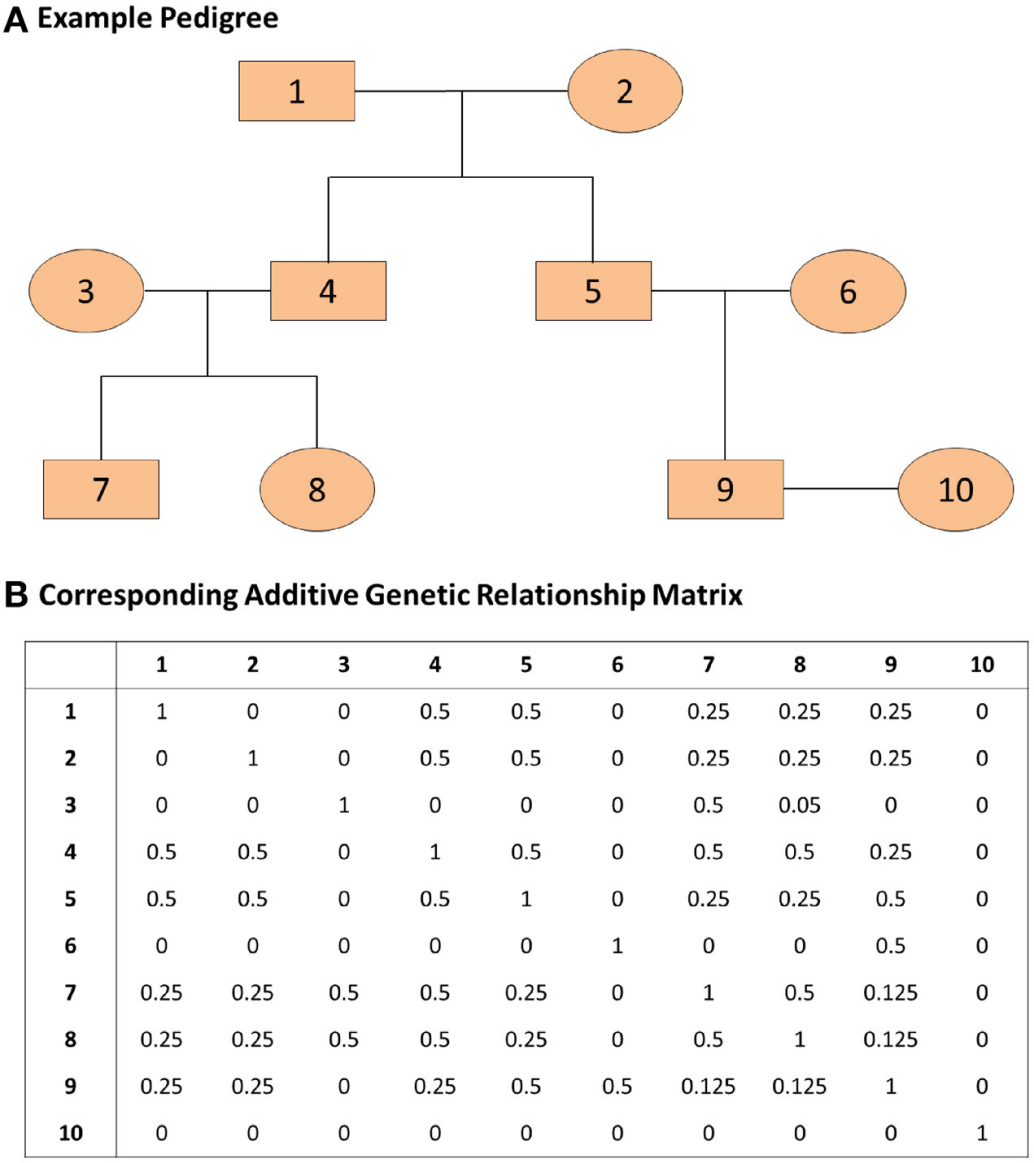
**An example pedigree and corresponding additive genetic relationship matrix**. **(A)** The pedigree on the top panel displays the relations among family members. **(B)** The additive genetic relationship matrix is the kinship matrix multiplied by 2; the kinship matrix contains kinship coefficients between any pair of family members and these coefficients represent the probability that two individuals share the same gene allele by identity by descent. The covariance between two family members *i* and *j* with kinship coefficient *k_ij_* is 2*k_ij_*σ^2^_*g*_ where σ^2^_*g*_ represents the genetic variance.

A common parameterization of linear mixed models for correlated family data is:

(1) $$y|\beta ,\sigma_{g}^{2},\sigma_{e}^{2}=X\beta +\rho +\epsilon ,$$

where *y* is the phenotype vector of size *n* × 1, *X* is the *n* × (*p* + 1) design matrix that contains values of measured covariates, β is the fixed effect vector of size *p* × 1, ρ is the random effect vector of size *n* × 1, which accounts for the additive polygenic effect, and ϵ is a vector of random errors of size *n* × 1. The vectors ρ and ϵ are marginally independent and follow distributions:

$$\begin{array}{l} \rho \sim N(\underline{0},\sigma_{g}^{2}A)\\ \epsilon \sim N(\underline{0},\sigma_{e}^{2}I_{n}), \end{array}$$

where *A* represents the known additive genetic relationship matrix (see Figure 1), σ^2^_*g*_ is the genetic variance, and σ^2^_*e*_ is the error variance (Eu-ahsunthornwattana et al., 2014). Under this parameterization the variance-covariance matrix of the observation *y* is the matrix:

(2) $$Var(y|\beta ,\sigma_{g}^{2},\sigma_{e}^{2})=\sigma_{g}^{2}A+\sigma_{e}^{2}I_{n}.$$

One can estimate the narrow-sense heritability of a trait as a ratio of the genetic variance σ^2^_*g*_ to the total phenotypic variance σ^2^_*g*_ + σ^2^_*e*_, such that $h^{2}=\frac{\sigma_{g}^{2}}{\sigma_{g}^{2}+\sigma_{e}^{2}}$.

In a recent review article, Muller et al. (2013) described the following parameterization for general linear mixed-effects models:

(3) $$y|\beta ,\sigma_{g}^{2},\sigma_{e}^{2}=X\beta +Gu+\epsilon$$

where *u* ~ *N*(0,σ^2^_*g*_*I_s_*), *e* ~ *N*(0, σ^2^_*e*_*I_n_*), and *G* is a matrix of known coefficients. The advantage of the parameterization in Equation (3) is that the random effects *u_i_*, *i* = 1, …, *n* are marginally independent rather than being correlated as in the initial parameterization.

The two parameterizations are equivalent once the matrix *G* is derived from the singular value decompostion of the matrix *A*. Specifically, by setting *A* = *US*^1/2^*S*^1/2^*U^T^* and letting *G* = *US*^1/2^, the variance-covariance matrix of *y* from the model in Equation (3) is

(4) $$\begin{array}{l} Var(y|\beta ,u,e)=\sigma_{g}^{2}GG^{T}+\sigma_{e}^{2}I_{n}=\sigma_{g}^{2}US^{1/2}S^{1/2}U^{T}+\sigma_{e}^{2}I_{n}\\ \;=\sigma_{g}^{2}A+\sigma_{e}^{2}I_{n} \end{array}$$

Note that the matrix *US*^1/2^ needs to be computed only once. We provide an example R script that computes *US*^1/2^ given a family-based data set in the Supplementary Material.

The parameterization can be extended to generalized linear mixed models. In a generalized linear mixed model (GLMM), the formulation becomes:

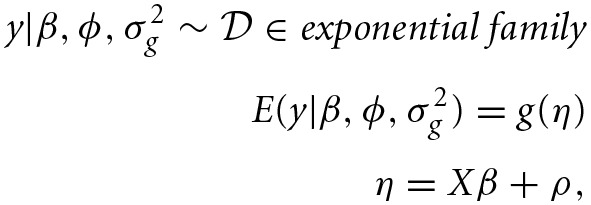



where ϕ is the dispersion parameter of the distribution belonging to the exponential family and *g*( · ) is the link function. The parameterization of the random effects applies as before and the linear predictors include the random effects in addition to the fixed effects. The difference here is the assumption that the observations are independent, conditionally on the random effects.

## 3. A real data example

As an example to illustrate the implementation in OpenBUGS, we consider the task of estimating the heritability of transferrin receptor levels from a large family-based study. The data are from the Long Life Family Study (LLFS) that between 2006 and 2009 enrolled approximately 5000 individuals from 583 families demonstrating clustering for longevity and healthy aging in the United States and Denmark (Sebastiani et al., 2009; Newman et al., 2011). A typical family of the LLFS includes a proband, the proband's siblings, their spouses, offspring of probands and siblings, and their spouses. The family size varies between 3 individuals to 77 individuals. In this example, transferrin receptor levels were adjusted for age at enrollment in the study and insulin levels. The kinship matrix *A* was calculated with the R package *kinship*2 (Therneau et al., 2012) and the R code to generate the matrix *G* is provided in Supplementary Material.

The entire BUGS code is shown with some comments below. There are a few points that are noteworthy. First, the matrix *G*, which is computed within R, is part of the BUGS data input. The calculation of the matrix *G* is required only once. Second, the variable offset is used to indicate the individuals who belong to each specific family. For example, in the code below, we use the index *i* to represents families and the index *j* to represent individuals. The first few values of the variable offset in this particular example are *c*(1, 8, 16, …). When *i* = 1 (the first family), *j* will span from 1 to 7, indicating that individuals 1 through 7 belong to family 1. When *i* = 2 (the second family), *j* will span from 8 to 15, indicating that individuals 8 through 15 belong to family 2. Then, based on the values of *j* and offset, the inner product between an appropriate row of the matrix *G* and vector *u* is computed.

A total of 11,000 iterations with the first 1000 as a burn-in was sufficient to reach the convergence of the estimates. On average, each iteration took 0.0892 s on an Intel(R) Core i5 processor (2.53 GHz) with 4 GB of RAM. The data were also analyzed using the method proposed in Hallander et al. (2010) and by fitting classical linear mixed models with the function lmekin() in *coxme* (Therneau, 2009) package in R.


The BUGS code
model svd {
   ## loop over families
   for( i in 1:n.fam) {
      ## loop over individuals within each
         family
      for(j in offset[i]:(offset[i+1]
      - 1) ){
      y[j]  ~  dnorm( mu[j], tau.e)
         mu[j] <- b0 + b.age*age[j] +
         b.insulin*insulin[j] +
         ## X *%* Beta
         inprod(G[j,offset[i]:(offset[i+1]-1)
         ], u[offset[i]:(offset[i+1]-1))
         ## G *%* u
      }
   }

## Model random effects as univariate normal
for( t in 1:N) { u[t]  ~ dnorm( 0, tau.g) }

## priors for fixed effects
b0  ~ dnorm(0,0.001)
b.age  ~ dnorm(0,0.001)
b.insulin  ~ dnorm(0,0.001)

## varance components
tau.e  ~ dgamma(1,   1)
tau.g  ~ dgamma(1 ,   1)
sigma.g2 <- 1/tau.g
sigma.e2 <- 1/tau.e
## narrow-sense heritability
herit <- (1/tau.g)/( 1/tau.g+1/tau.e)
}


**Figure 2:**
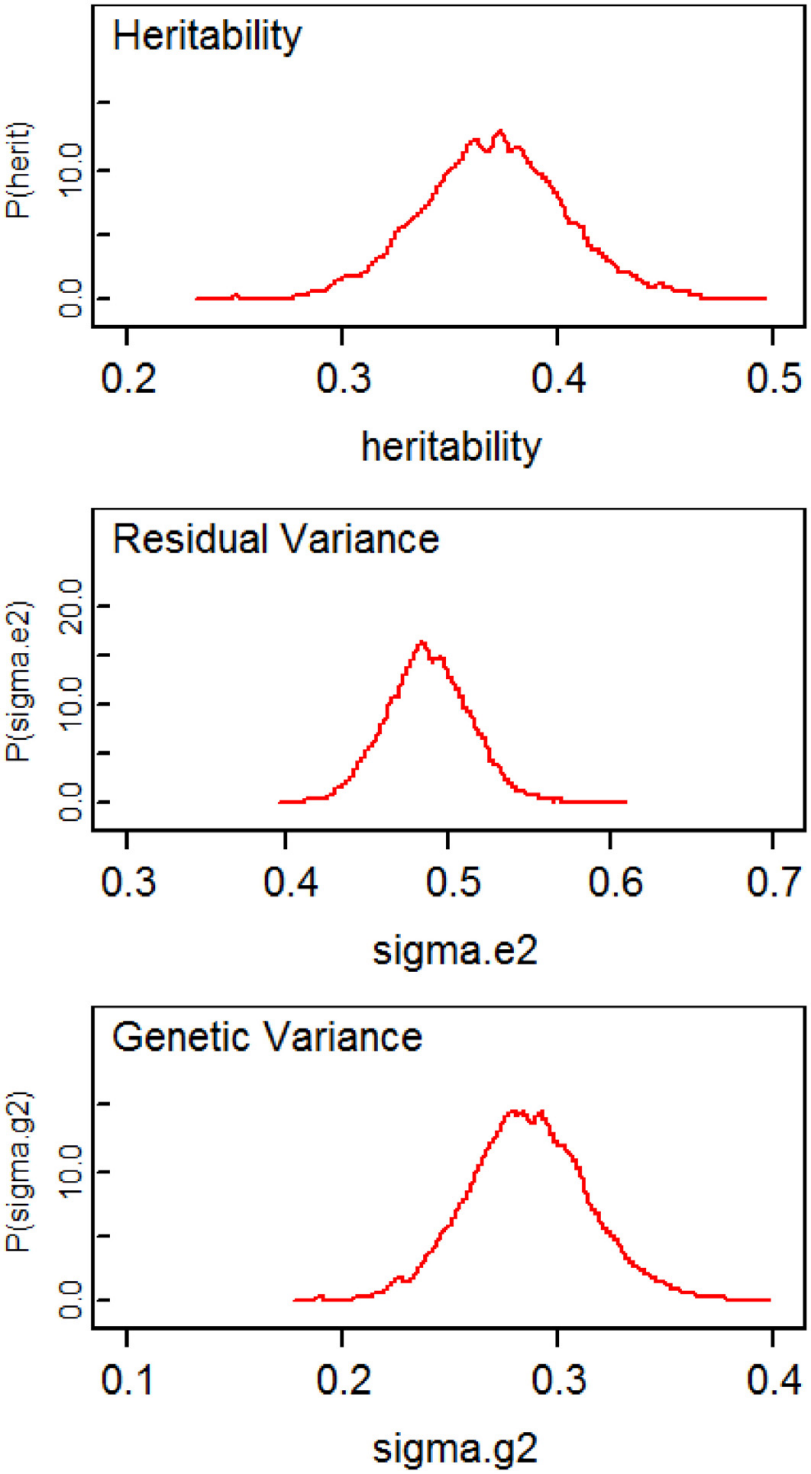
**Plots of posterior distributions of heritability, residual variance, and genetic variance**.

Table 1 compares the point estimates and standard errors (*SE*) of regression parameters, the variance parameters, and the heritability estimates from the linear mixed models computed using the lmekin() function in R, the proposed method (SVD Model), and the method in Hallander et al. (2010) (conditional Model). Figure 2 displays the posterior distribution of heritability, residual variance, and genetic variance. The point estimates and SE from the R ouput and SVD model are nearly identical. The heritability estimates in the two methods are 0.3677 and 0.3677, respectively, with a difference of only 0.0031. However, compared to these two methods, the conditional model produces discrepant results; the residual variance is over-estimated and the genetic variance is under-estimated, which leads to inconsistent estimate of the heritability and the 95% credible intervals of the two Bayesian methods do not overlap. Inconsistent results between the SVD model and conditional model are surprising since, in theory, both approaches rely on decomposition methods that should lead to exactly the same covariance matrix. To further investigate this discrepancy, simulations of several scenarios of extended pedigree data structures were performed (see the next section for details on simulations). However, we were not able to pinpoint the reason for the apparent discrepancy. The advantage of the Bayesian approach here, compared to the classical approach is to provide measures of the uncertainity of the heritability estimate by the posterior distribution (Figure 2) and the 95% credible interval.

**Table 1:** **Comparison of Point Estimates (*PE*), standard errors (*SE*), and 95% Credible Intervals (95% *CI*) for continuous trait**.

	**R (lmekin)**		**SVD model**			**Conditional model**		
	***PE***	***SE***	***PE***	***SE***	**95%*CI***	***PE***	***SE***	**95% *CI***
Intercept	2.1494	0.0652	2.143	0.0697	2.014–2.283	2.153	0.0730	2.018–2.3
Age	0.0101	0.0008	0.0102	0.0009	0.0084–0.0119	0.0101	0.0009	0.0082–0.0119
Insulin	0.0021	0.0004	0.0022	0.0004	0.0014–0.0030	0.0022	0.0004	0.0014–0.0030
Heritability	0.3677	N/A	0.3707	0.0345	0.3015–0.4402	0.1325	0.0257	0.0957–0.195
Residual variance	0.4877	N/A	0.4866	0.0263	0.436–0.5402	0.6624	0.02439	0.6114–0.7074
Genetic variance	0.2837	N/A	0.2862	0.0289	0.2303–0.3466	0.1013	0.0198	0.0733–0.1494

*R (lmekin), results obtained from using lmekin function in R; SVD Model, results obtained from using the proposed method based on singular value decomposition of the additive genetic relationship matrix; Conditional Model, results obtained from using the method in Hallander et al. (2010). The total sample size was 4229 with 558 unique families*.

We extended our approach to logistic regression with family data. To our knowledge, no statistical package fully adjusts for the familiar relatedness when the outcome variable is binary. A commonly used approach is to fit logistic regression with one random effect per family or use a generalized estimating equation (GEE), in which each family is considered a cluster. Thus, there is a need to develop such methods to analyze binary traits coming from family data. As an example, we again used the data from the LLFS, in which the binary trait was the occurrence of cardiovascular diseases within 8 years of follow-up and covariates were sex and age at enrollment of participants. Cardiovascular diseases were defined as having any one of the following: myocardial infarction, coronary artery bypass grafting, congestive heart failure, and atrial fibrillation (Sebastiani et al., 2013). We modified the BUGS code by changing the response variable *y*[*j*] to follow a Bernoulli distribution and modeling the random effects vector on the log-odds scale. For comparison, a logistic regression model based on the GEE approach was also performed. Table 2 shows the point estimates, standard errors, and 95% credible intervals based on the two approaches. The point estimates between the two methods are comparable, although the standard errors from the GEE model are slightly smaller for all three fixed effects parameters. This is expected, as our proposed model takes into account the full kinship matrix, whereas the GEE model treats each family as a single cluster. It is also noteworthy to point out that convergence can be slow for implementing this parameterization in a logistic regression framework. A good heuristic for faster convergence is to start with the maximum likelihood estimates of the fixed effects parameters and then try to estimate the genetic variance σ^2^_*g*_.

**Table 2:** **Comparison of Point Estimates (*PE*), Standard Errors (*SE*), and 95% Credible Intervals (95% *CI*) for binary trait**.

	**SVD model**			**GEE model**		
	***PE***	***SE***	**95% *CI***	***PE***	***SE***	**95% *CI***
Intercept	−5.186	0.231	−5.646–−4.742	−5.023	0.210	−5.430–−4.610
Sex	−0.470	0.077	−0.621–−0.318	−0.458	0.075	−0.604–−0.311
Age	0.064	0.0026	0.058–0.069	0.062	0.0024	0.057–0.066

*SVD Model, results obtained from using the proposed method based on singular value decomposition of the additive genetic relationship matrix in a logistic regression; GEE Model, results from generalized estimating equations in a logistic regression. The total sample size was 4654 with 583 unique families*.

## 4. Empirical evaluation

A simulation study was conducted to evaluate the accuracy of the implementation of our method in different types of family structure for normal data. Four different scenarios, in which the current implementation was evaluated, are as follows:

- *Nuclear Family*: This is the simplest family structure in which there is a couple with two offspring. There were a total of 100 such families, which led to *N* = 400.
- *Two-trios*: This is the simplest form of extended pedigree structure with first-, second-, and third-degree relatives where two parent-offspring trios are related through a sibling pair in the parent generation. There were a total of 100 such families and the total sample size was 600.
- *Asymmetric Family*: This is an asymmetric version of the second scenario, in which the first trio has only one offspring and the second trio has ten offspring. There were a total of 100 such families with a total sample size of 1500.
- *Combination*: This is a combination of the first and second scenario with several offspring in the second scenario. The total sample size was 1400.

To generate correlated data, a kinship matrix from each family *K_f_* was created, and the variance-covariance matrix of the observations was defined as *V* = σ^2^_*e*_*I_n_* + 2 σ^2^_*g*_ *diag*(*K*_1_, *K*_2_, …, *K_n_f__*), where *n* is the total sample size and *n_f_* is the number of families in each scenario. In each simulation, a vector *Z* of independent and normally distributed observations was generated and transformed into *Y* = *UD*^1/2^*Z* where *U* and *D* are the matrix of eigenvectors and eigenvalues from the spectral decomposition of the variance-covariance matrix *V*. This transformation guarantees that *V*(*Y*) = *UD*^1/2^*V*(*Z*)*D*^1/2^*U^T^* = *V* so that the simulated data have the desired correlation.

Table 3 compares the point estimates and standard errors of the variance components from the linear mixed models computed using the lmekin() function in R, the proposed method (SVD Model), and the method in Hallander et al. (2010) (conditional Model). In all scenarios, there was no discernible difference between the estimates among the three approaches, which suggests that the implementation in BUGS works correctly.

**Table 3:** **Comparison of Point Estimates (*PE*) and Standard Errors (*SE*) of variance components in simulated data**.

		**R (lmekin)**		**SVD model**		**Conditional model**	
		***PE***	***SE***	***PE***	***SE***	***PE***	***SE***
Nuclear family	σ^2^_*e*_	1.214	N/A	1.220	0.186	1.217	0.181
	σ^2^_*g*_	0.734	N/A	0.738	0.211	0.741	0.206
Two-trios	σ^2^_*e*_	0.936	N/A	0.926	0.193	0.936	0.200
	σ^2^_*g*_	1.938	N/A	1.963	0.279	1.944	0.287
Asymmetric family	σ^2^_*e*_	0.963	N/A	0.961	0.090	0.956	0.087
	σ^2^_*g*_	1.024	N/A	1.029	0.143	1.043	0.141
Combination	σ^2^_*e*_	1.030	N/A	1.028	0.117	1.031	0.123
	σ^2^_*g*_	1.915	N/A	1.927	0.180	1.923	0.186

*R (lmekin), results obtained from using lmekin function in R; SVD Model, results obtained from using the proposed method based on singular value decomposition of the additive genetic relationship matrix; Conditional Model: results obtained from using the method in Hallander et al. (2010)*.

## 5. Conclusion

The proposed BUGS code provides an easy and efficient way to account for extended family structures in linear mixed models. Results from a real data set as well as simulation data show that this implementation produces consistent results with the classical linear mixed models in R. The usefulness of this approach is that it allows for linear mixed modeling of family-based data in the BUGS software, and thus possibly facilitates the use of Bayesian modeling of family-based data. The advantage of the Bayesian approach is that it provides an estimate of heritability but implementation is often challenging. We also illustrate the extension of our approach to generalized linear models that can be efficiently implemented in BUGS.

### Conflict of interest statement

The authors declare that the research was conducted in the absence of any commercial or financial relationships that could be construed as a potential conflict of interest.

## Supplemental data

Sample R script for computing the singular value decomposition of the additive genetic relationship matrix.

## Read the Phenotype Data ##
pheno.file <- read.csv("pheno.data.csv")

## Read the Pedigree Data
ped.file <- read.csv("ped.data.csv")

## Variable Description:
## subject = unique individual ID
## gpedid = family ID
## dadsubj = ID of the father
## momsubj = ID of the mother
## sex = sex of individual

## Needs to be ordered by family
ped.file <- ped.file[ order(ped.file$pedid),]

## Use package kinship2 to construct the pedigree
   object and
## the kinship matrix.
library(kinship2)
## This creates pedigree objects
mped.full <- pedigree(id=ped.file$subject,
dadid=ped.file$dadsubj,
momid=ped.file$momsubj, sex=ped.file$sex,
famid=ped.file$gpedid,missid=0)
## This creates the kinship matrix
kmat.full <- kinship(mped.full)

## Singular Value Decomposition by family
family.list <- intersect(unique(ped.file$gpedid),
unique(pheno.file$gpedid))
my.U <- vector("list")
my.S <- vector("list")
my.kmat <- vector("list")
my.G <- vector("list")

for(i in 1:length(family.list)){
   p <- ped.file[ which(ped.file$gpedid
   == family.list[i]),]
   mped <- pedigree(id=p$subject, dadid=p$dadsubj,
                momid=p$momsubj,sex=p$sex,famid
                =p$gpedid,missid=0)
kmat <- 2*kinship(mped)
## Now get kinship submatrix for subjects
ind.subj <- match(pheno.file$subject
                 [which(pheno.file$gpedid ==
                 family.list[i])], row.names(kmat))
test <- svd( kmat[ind.subj[ which(is.na
                 (ind.subj)==F)],
             ind.subj[ which(is.na(ind.subj)==F)]])
my.U[[i]] <- test$u
my.S[[i]] <- test$d
my.G[[i]] <- test$u %*% diag( sqrt(test$d))
my.kmat[[i]] <- kmat[ind.subj
                    [ which(is.na(ind.subj)==F)],
            ind.subj[ which(is.na(ind.subj)==F)]]
}

## This is the matrix to be loaded to bugs
G.mat <- my.G[[1]]
for(i in 2:length(my.G)){
   ith.block.1 <- rep(0, nrow(G.mat)*ncol(my.G[[i]]))
   dim(ith.block.1) <- c(nrow(G.mat), ncol(my.G[[i]]))
   ith.block.2 <- rep(0, ncol(G.mat)*nrow(my.G[[i]]))
   dim(ith.block.2) <- c(nrow(my.G[[i]]), ncol(G.mat) )
   G.mat <- rbind( cbind(   G.mat, ith.block.1),
                cbind(ith.block.2, my.G[[i]]))
}
